# Redox‐regulated signalling of adaptations to contractile activity in skeletal muscle: Implications for age‐related muscle weakness

**DOI:** 10.1113/EP092458

**Published:** 2025-08-05

**Authors:** Malcolm J. Jackson

**Affiliations:** ^1^ Department of Musculoskeletal and Ageing Science, Institute of Life Course and Medical Sciences University of Liverpool Liverpool UK

**Keywords:** hydrogen peroxide, mitochondria, motor neuron, NMJ

## Abstract

Skeletal muscle adaptation to contractile activity is modulated by redox signalling, primarily through reactive oxygen species (ROS) such as hydrogen peroxide (H_2_O_2_). Early research framed ROS as deleterious byproducts of exercise, but subsequent studies have established their roles as signalling molecules involved in mitochondrial biogenesis, stress responses and metabolic regulation. Central to this process appear to be peroxiredoxins (Prdxs), particularly Prdx2, which current evidence suggests mediate redox relays by sensing physiological H_2_O_2_ levels and initiating transcriptional programs. Our recent findings demonstrate that low levels of H_2_O_2_, or electrically induced contractions, rapidly oxidise Prdx1, Prdx2 and Prdx3 in mouse muscle fibres. Transcriptomic analysis of human skeletal muscle myotubes confirmed that Prdx2 is essential for upregulating mitochondrial genes in response to H_2_O_2_ or contraction. With ageing, skeletal muscle exhibits impaired redox signalling with elevated ROS levels. Using an ageing mouse model, we observed diminished Prdx2 oxidation during contraction, suggesting redox signalling dysfunction. This impaired response likely contributes to sarcopenia by blunting the adaptive capacity of aged muscle. Our findings emphasise the importance of redox homeostasis (not merely ROS suppression) in maintaining muscle health. Understanding the nuanced role of ROS and Prdxs in exercise adaptation and ageing could inform therapeutic strategies aimed at restoring redox‐sensitive signalling to preserve muscle function across the lifespan.

## Abstract (highlights)

- **What is the topic of this review?**
  The role of redox signalling in adaptations of skeletal muscle to contractile activity and how this is modified by ageing.
- **What advances does it highlight?**
  The potential role of peroxiredoxins as mediators of redox signalling in muscle.

## 1 INTRODUCTION

Early research in the late 1970s and early 1980s proposed that exercise induces an increase in reactive species, particularly free radicals, within muscle tissue, leading to lipid peroxidation (Brady et al., 1979; Davies et al., 1982; Dillard et al., 1978; Gee & Tappel, 1981; Jackson et al., 1983a, 1983b; Jackson et al., 1985). At the time, these reactive species were largely viewed as harmful due to their ability to oxidise key cellular components such as lipids, proteins and nucleic acids (Halliwell & Gutteridge, 1984). Consequently, numerous early investigations sought to mitigate their damaging effects, frequently through antioxidant supplementation, especially with vitamin E (Brady et al., 1979; Jackson et al., 1983b). This perception evolved significantly with the work of Sen and Packer (1996) and others, who highlighted the physiological roles of reactive oxygen species (ROS) as signalling molecules. Since then, research has increasingly focused on the role of ROS in cellular signalling processes.

### 1.1 Redox signalling and muscle adaptations to contractions

Contractile activity in muscle fibres triggers an increase in intracellular superoxide and nitric oxide (NO) production, which subsequently leads to the formation of ROS and reactive nitrogen species (Palomero et al., 2008; Powers & Jackson, 2008; Pye et al., 2007). These reactive species activate multiple transcription factors, including nuclear factor‐κB (NF‐κB), activator protein 1 (AP‐1) and heat shock factor 1 (HSF‐1) (Jackson et al., 2002; Ji et al., 2004; Ristow et al., 2009; Vasilaki, McArdle et al., 2006), resulting in enhanced expression of regulatory enzymes and cytoprotective proteins (Hollander et al., 2003; McArdle et al., 2001; McArdle, Spiers et al., 2004). Genes involved in proteolysis (Bar‐Shai et al., 2005; Peterson & Guttridge, 2008; Van Gammeren et al., 2009) and mitochondrial biogenesis (Bakkar et al., 2008; Irrcher et al., 2009) are also subject to redox‐regulation.

The mechanism by which ROS influence signalling is largely through specific oxidative modifications of protein residues (Janssen‐Heininger et al., 2008; Sobotta et al., 2015). Despite growing evidence of these roles, pinpointing the precise redox‐regulated steps in skeletal muscle adaptation to exercise remains challenging. Studies employing antioxidant supplementation in both animals and humans have shown that high doses of antioxidants can dampen exercise‐induced adaptive responses, such as the upregulation of heat shock proteins (Venditti et al., 2014), mitochondrial biogenesis (Gomez‐Cabrera et al., 2008; Paulsen et al., 2014; Ristow et al., 2009), enhanced insulin sensitivity (Ristow et al., 2009) and cytokine release (Wuyts et al., 2003). Although the use of antioxidant supplements in this context remains debated (Gomez‐Cabrera et al., 2012; Higashida et al., 2011), further insights have emerged from genetically modified mouse models lacking ROS‐generating enzymes. For instance, NADPH oxidase 2 (Nox2)‐deficient mice exhibited reduced glucose uptake after exercise due to impaired glucose transporter type 4 (GLUT4) translocation (Henriquez‐Olguin, Renani et al., 2019), and mice lacking Nox4 developed insulin resistance (Xirouchaki et al., 2021). Moreover, endothelial‐specific Nox4 deletion hindered metabolic adaptations to long‐term exercise (Specht et al., 2021). These findings support the idea that ROS are integral to a wide array of exercise‐induced adaptations, and several signalling pathways linked to these adaptations are known to be redox‐sensitive (Jackson et al., 2020).

### 1.2 Physiological versus experimental H_2_O_2_ concentrations and the redox relay concept

A persistent challenge in elucidating redox‐regulated mechanisms in muscle is the disparity between physiological intracellular H_2_O_2_ levels and the higher concentrations typically used in in vitro studies to activate redox‐sensitive signalling pathways. While in vitro experiments often employ H_2_O_2_ concentrations between 10^−4^ and 10^−3^ M, cytosolic levels in resting muscle cells are generally in the 10^−9^ to 10^−8^ M range (Sies, 2017). Upon contraction, this may transiently rise to approximately 10^−7^ M (Jackson & McArdle, 2011; Sies, 2014), though this estimate may be somewhat inflated due to newer analyses of H_2_O_2_ kinetics (Huang & Sikes, 2014).

To resolve this inconsistency, the concept of redox relays has been proposed, in which thiol peroxidases, particularly 2‐Cys peroxiredoxins (Prdx), act as intermediates by reacting with H_2_O_2_ and transferring oxidising equivalents to downstream proteins (Stöcker et al., 2018). Prdx are well‐known as antioxidant enzymes that reduce peroxides, protecting cells from oxidative damage and potentially regulating redox signalling. Structurally, they typically form homodimers or higher‐order oligomers and use a conserved cysteine residue for catalysis. Six isoforms exist in mammals (Prdx1–6) (Chae et al., 2012). The Prdxs are subdivided into three types, depending on the number of redox‐active cysteine residues they contain: the typical 2‐Cys (Prdx1–4), atypical 2‐Cys (Prdx5) and 1‐Cys (Prdx6) types (Chae et al., 2012; Zhou et al., 2024). Prdxs are found in various cellular compartments and have exceptionally high reactivity toward H_2_O_2_, far exceeding that of many other redox‐sensitive proteins (Wood et al., 2003). A summary of the sub‐cellular location of the different mammalian Prdxs is shown in Table 1.

**TABLE 1:** Mammalian peroxiredoxin distribution and primary reductants when acting as antioxidants.

Peroxiredoxin	Prdx type	Sub‐cellular location	Reductant
Prdx1	2‐Cys	Nuclei, cytosol	Trx1
Prdx2	2‐Cys	Nuclei, cytosol	Trx1
Prdx3	2‐Cys	Mitochondria	Trx2
Prdx4	2‐Cys	Cytosol, SR/ER	Trx1
Prdx5	Atypical 2‐Cys	Cytosol, mitochondria, peroxisomes	Trx1, Trx2
Prdx6	1‐Cys	Cytosol	GSH

*Note*: Data derived from Chae et al. (2012) and Zhou et al. (2024).

Several studies have confirmed that Prdxs can directly oxidise target proteins via heterodimer formation, initiating signalling cascades (Jarvis et al., 2012; Sobotta et al., 2015; Talwar et al., 2020). This positions Prdxs as critical mediators connecting H_2_O_2_ production during contraction with activation of redox‐responsive pathways.

Our previous findings demonstrated that even brief exposure of mature isolated mouse muscle fibres to low extracellular concentrations of H_2_O_2_ (as low as 2.5 µM), or a short period of isometric contraction, induces oxidation and dimerisation of Prdx1, Prdx2 and Prdx3. Notably, Prdx2 oxidation was detectable within just 1 min following 12 contractions (Stretton et al., 2020). These results suggest that these Prdx isoforms may serve as redox sensors during muscle contraction. The extremely rapid oxidation of Prdx2 suggests this isoform is particularly sensitive to contraction‐induced oxidation, but particularly intriguing was the oxidation of mitochondrial Prdx3 following contractile activity. Recent evidence has generally supported the concept that the intramuscular ROS produced during such contractions primarily originate from cytosolic NADPH oxidases (Henriquez‐Olguin, Knudsen et al., 2019; Sakellariou et al., 2013), but the oxidation of Prdx3 seen in these studies provides indirect evidence that additional mitochondria‐localised sources of ROS are activated during contractions. This has been suspected for a considerable time (Davies et al., 1982; Jackson et al., 1985), but there has been a lack of direct evidence to support this possibility (Henriquez‐Olguin, Boronat et al., 2019; Sakellariou et al., 2013).

### 1.3 Transcriptomic evidence linking H_2_O_2_, Prdx2 and muscle gene expression

**FIGURE 1:**
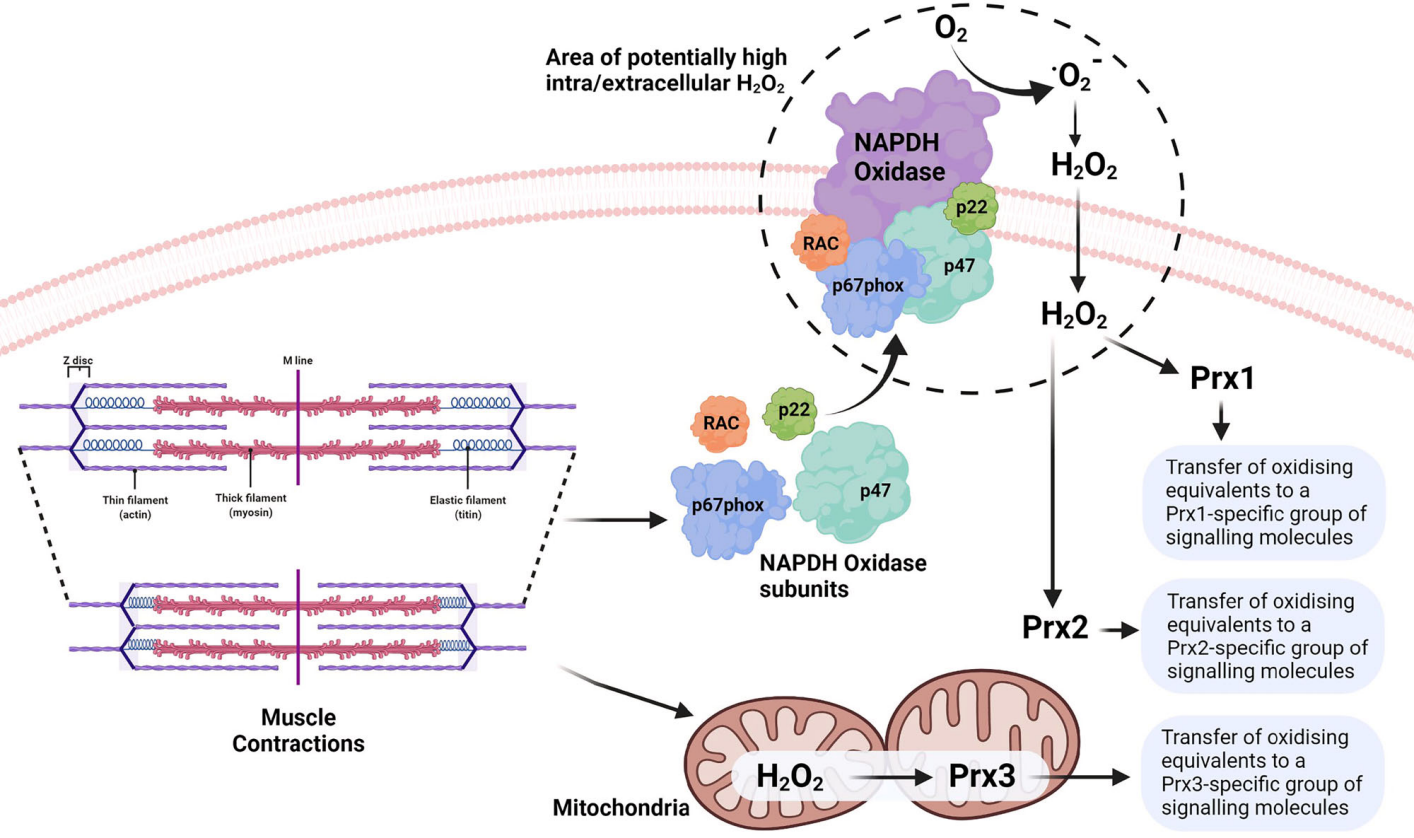
Potential mechanisms underlying generation of ROS (superoxide and H_2_O_2_) during muscle contractions and oxidation of 2‐Cys peroxiredoxins in different cellular compartments, leading to activation of specific groups of peroxiredoxin‐interacting signalling molecules involved in muscle adaptations to the contractile activity. Figure reproduced from Jackson et al. (2022) with peroxiredoxins denoted as Prx (referring to mouse protein).

Supporting this study on mouse muscle fibres, recent work in *Caenorhabditis elegans* by Xia et al. (2023) showed that loss of Prdx2 impaired exercise‐induced adaptations during swimming activity. In order to further examine the potential role of Prdx2, we recently conducted a study utilising RNA sequencing in cultured human skeletal muscle myotubes (Heaton et al., 2025). We observed a high degree of overlap in differentially expressed genes (DEGs) following either 15 min of electrically stimulated isometric contraction or exposure to low concentrations (2.5 or 5 µM) of H_2_O_2_. Commonly upregulated genes across conditions included those involved in mitochondrial oxidative phosphorylation, such as *COX1*, *COX2*, *COX3* and *ATP6*. However, this upregulation was absent in myotubes with Prdx2 knockdown (Prdx2KD), suggesting that Prdx2 is essential for translating H_2_O_2_ signalling into mitochondrial gene expression. These findings collectively support the view that both contractile activity and physiological levels of H_2_O_2_ promote the expression of mitochondrial oxidative phosphorylation genes in human skeletal muscle, and that Prdx2 plays a pivotal role in mediating this redox‐driven adaptation. A schematic overview of the current understanding of H_2_O_2_ and Prdx‐mediated redox regulation in muscle adaptation is provided in Figure 1.

### 1.4 Disruption of redox signalling and skeletal muscle adaptation during ageing

As individuals age, a decline in skeletal muscle mass and strength contributes significantly to instability, increased risk of falls and loss of independence (Young & Skelton, 1994). By the age of 70, skeletal muscle cross‐sectional area typically declines by 25–30%, accompanied by a 30–40% reduction in muscle strength (Porter et al., 1995). This phenomenon, referred to as sarcopenia, arises from both the loss of muscle fibres and the atrophy and weakening of those that remain (Brooks & Faulkner, 1988; Lexell et al., 1986, 1988). Concomitantly, the number of motor units also decreases with age in both humans and rodent models (Campbell et al., 1973; Sheth et al., 2018), with studies showing a 25–50% reduction in motor neurons (Rowan et al., 2012; Tomlinson & Irving, 1977). Our research has demonstrated that approximately 15% of muscle fibres in aged mice are entirely denervated, and up to 80% of neuromuscular junctions (NMJs) display structural abnormalities (Vasilaki et al., 2016).

Ageing is also characterised by a diminished ability to adapt to physiological stress (Pomatto & Davies, 2017). In skeletal muscle, this manifests partly as a blunted response to exercise‐induced stress, impaired mitochondrial biogenesis and reduced anabolic signalling (Cuthbertson et al., 2005; Vasilaki, McArdle et al., 2006; Viña et al., 2009).

Contrary to early assumptions, ROS levels or oxidative damage alone do not directly determine lifespan. Nevertheless, altered ROS activity has been implicated in the progression of age‐associated pathologies (Hamilton et al., 2012). Ageing skeletal muscle exhibits elevated mitochondrial ROS production, including hydrogen peroxide generated by mitochondria (Vasilaki, Mansouri et al., 2006a).

To investigate the role of ROS in muscle ageing, genetically modified mouse models lacking specific ROS‐regulating enzymes have been studied. Although these models often display increased oxidative stress, clear links to accelerated muscle ageing are rare (Jang & Van Remmen, 2009). An exception is the whole‐body superoxide dismutase 1 knockout (Sod1KO) mouse, which presents with elevated oxidative damage, motor neuron degeneration and premature sarcopenia‐like features (Deepa et al., 2019; Muller et al., 2006). The Sod1KO mouse lacks Sod1 in all tissues, but this pure Sod1 knockout model does not show the symptoms of motor neuron disease (ALS), which occur in multiple mouse models with mutations in *Sod1*, such as the SOD1(G93A) mouse model (Peggion et al., 2022). In the Sod1KO mouse, significant declines in muscle mass, motor unit number, contractile strength and mitochondrial function occur by 8 months of age (Jang et al., 2010; Larkin et al., 2011; Vasilaki et al., 2010) and degeneration of NMJs in Sod1KO mice parallels that observed in aged wild‐type mice but occurs much earlier.

Sod1KO mice also display diminished redox‐responsive adaptation to contractile activity, mirroring what is seen in naturally aged muscle (Vasilaki et al., 2010). Since Sod1's sole known function is catalysing the conversion of superoxide to hydrogen peroxide, its absence suggests broader consequences for redox homeostasis. Superoxide can also interact rapidly with nitric oxide to form peroxynitrite – a process even faster than its dismutation to hydrogen peroxide. Given that nitric oxide concentrations are higher than superoxide in muscle, this reaction becomes particularly relevant when Sod is deleted. Elevated peroxynitrite levels in Sod1KO muscle may underlie the observed acceleration of muscle degeneration (Sakellariou et al., 2011).

Importantly, the early onset of sarcopenia‐like symptoms in Sod1KO mice largely stems from impairments at the motor neuron level. Studies using neuron‐specific Sod1 deletion (i‐mnSod1KO) (which also shows no symptoms of motor neurone disease) have confirmed that although the deletion does not induce unique effects, it accelerates age‐related degenerative changes. These include reduced axonal size, increased denervation at NMJs, simplified acetylcholine receptor morphology, and structural abnormalities in nerves and NMJs – all of which resemble changes in much older wild‐type mice (Pollock et al., 2023).

While Sod1 deficiency does not occur naturally with age, the phenotypic similarities between Sod1KO and aged wild‐type mice validate the use of this model in studying age‐related muscle decline. The data collectively indicate that age‐related disruptions in ROS generation and impaired redox signalling contribute to reduced muscle adaptability to exercise. Genetic interventions targeting these signalling deficiencies have shown promise in mitigating some of the adverse effects of ageing in animal models (Kayani et al., 2010; McArdle, Dillmann et al., 2004).

Our research has connected increased mitochondrial hydrogen peroxide production in aged and Sod1KO mice to a suppression of redox‐sensitive signalling. We hypothesise that this ROS elevation induces upregulation of antioxidant enzymes like peroxiredoxins (Prdx), glutathione peroxidases (GPx) and thioredoxins (TrX), which may then buffer ROS too efficiently, preventing critical cysteine oxidation in signalling proteins (Jackson, 2020). In addition, recurrent cycles of partial denervation and reinnervation – common during ageing – may contribute to disrupted mitochondrial ROS dynamics and impair mitochondrial integrity and function (Jackson, 2020; Scalabrin et al., 2019).

To directly assess the impact of age on redox signalling, our research group examined peroxiredoxin oxidation in isolated contracting mouse flexor digitorum brevis (FDB) fibres (Stretton et al., 2020). The data obtained are summarised in Table 2. Ageing did not significantly modify the pattern of Prdx1 or mitochondrial Prdx3 oxidation, but the extent and time course of cytosolic Prdx2 oxidation were significantly modified in old mice during contractions, and the level of oxidation did not reach that achieved in adult mice at any point within the 15‐min contraction protocol. This suggests impaired redox signalling and reduced activation of transcriptional responses (Cobley et al., 2014) may be linked to modified Prdx2 oxidation, reinforcing the idea that Prdxs play a crucial role in initiating exercise‐induced signalling. Notably, Prdx2 oxidation at rest was also lower in aged muscle, despite reports of increased ROS levels in these tissues (Palomero et al., 2013; Sastre et al., 2003; Vasilaki, Mansouri et al., 2006).

**TABLE 2:** Summary of the effect of ageing on levels of oxidation of 2‐Cys peroxiredoxins induced by contractile activity in mouse FDB fibres.

	Adult	Old
**Peroxiredoxin 1 oxidation**	Significantly increased by 2 min of contractions	No significant change at rest compared with adult; significantly increased by 2 min of contractions
**Peroxiredoxin 2 oxidation**	Significantly increased by 1 min of contractions	Significantly reduced oxidation at rest compared with adult; significantly increased by 15 min of contractions
**Peroxiredoxin 3 oxidation**	Significantly increased by 2 min of contractions	No significant change at rest compared with adult; significantly increased by 2 min of contractions

*Note*: Data derived from Stretton et al. (2020).

This seemingly contradictory observation may be explained by the concept of ‘molecular habituation’, where chronic exposure to a stimulus diminishes the responsiveness of downstream signalling via upregulation of inhibitory regulators (Martinez Guimera et al., 2018). One specific candidate is thioredoxin‐1, whose levels are elevated in aged muscle and may suppress Prdx oxidation (Dimauro et al., 2012), but ageing in mice has also been associated with an increase in the skeletal muscle content and activities of other antioxidant enzymes, such as glutathione peroxidase and catalase (Palomero et al., 2013), Prdx3 and Prdx6 (McDonagh et al., 2014)

## 2 CONCLUSIONS

The evidence reviewed here strongly supports the idea that disrupted redox signalling contributes to the decline in skeletal muscle mass and function with age. In particular, ageing is associated with increased mitochondrial ROS production, altered antioxidant enzyme activity and impaired redox‐sensitive responses to contractile activity. These changes limit the muscle's ability to adapt to physiological stress such as exercise, which may contribute to the progression of sarcopenia. The Sod1KO mouse model, despite its genetic specificity, effectively mimics many of the hallmarks of age‐related muscle degeneration seen in wild‐type animals, including NMJ degradation, motor unit loss and blunted redox signalling. These parallels have made the model a valuable tool for exploring the mechanisms behind muscle ageing, especially in understanding how oxidative stress and redox imbalance affect neuromuscular health. Moreover, emerging concepts such as molecular habituation may explain why aged muscle appears less responsive to ROS‐driven signalling despite elevated oxidative stress. The upregulation of antioxidant systems like thioredoxins may paradoxically suppress beneficial redox‐mediated signalling events by over‐buffering the intracellular environment.

Collectively, these findings highlight the complexity of redox regulation in ageing muscle and suggest that therapeutic strategies aimed at restoring proper redox signalling – rather than simply reducing ROS levels – could be more effective in preserving muscle function and promoting healthy ageing.

## AUTHOR CONTRIBUTIONS

Sole author.

## CONFLICT OF INTEREST

None declared.

## FUNDING INFORMATION

No funding was received for this work.
